# The potential of tRF-21-U0EZY9X1B plasmatic level as a biomarker of children with obstructive sleep apnea-hypopnea syndrome

**DOI:** 10.1186/s12887-023-04020-2

**Published:** 2023-04-26

**Authors:** Yanbo Lu, Qiang Fu, Xiaohong Cai, Yijing Shen, Junhua Wu, Haiyan Qiu

**Affiliations:** 1 Medical School of Ningbo University, Ningbo, Zhejiang China; 2 Ningbo Women and Children’s Hospital, Ningbo, Zhejiang China; 3 Jingzhou Hospital Affiliated to Yangtze University, Jingzhou, Hubei China

**Keywords:** Children, Obstructive sleep apnea-hypopnea syndrome, tRNA-derived fragments, Biomarker

## Abstract

**Purpose:**

We investigated changes in plasma transfer RNA related fragments (tRF) in children with obstructive sleep apnea–hypopnea syndrome (OSAHS) and the potential value as a disease marker.

**Methods:**

Firstly, we randomly selected five plasma samples from the case group and the control group for high-throughput RNA sequencing. Secondly, we screened one tRF with different expression between the two groups, amplified it by quantitative reverse transcription-PCR (qRT-PCR) and sequenced the amplified product. After confirming that the qRT-PCR results were consistent with the sequencing results and the sequence of the amplified product contained the original sequence of the tRF, we performed qRT-PCR on all samples. Then we analyzed the diagnostic value of the tRF and its correlation with some clinical data.

**Results:**

A total of 50 OSAHS children and 38 control children were included in this study. There were significant differences in height, serum creatinine (SCR) and total cholesterol (TC) between the two groups. The plasma expression levels of tRF-21-U0EZY9X1B (tRF-21) were significantly different between the two groups. Receiver operating characteristic curve (ROC) showed that it had valuable diagnostic index, with area under the curve (AUC) of 0.773, 86.71% and 63.16% sensitivity and specificity.

**Conclusions:**

The expression levels of tRF-21 in the plasma of OSAHS children decreased significantly which were closely related to hemoglobin, mean corpuscular hemoglobin, triglyceride and creatine kinase-MB, may become novel biomarkers for the diagnosis of pediatric OSAHS.

**Supplementary Information:**

The online version contains supplementary material available at 10.1186/s12887-023-04020-2.

## Introduction

Obstructive sleep apnea hypopnea syndrome (OSAHS) is a sleep disorder caused by partial or complete obstruction of the upper respiratory tract, accompanied by apnea and insufficient ventilation during sleep [1]. It is a common chronic disease in children. The prevalence of OSAHS in children ranged from 1.0 to 5.7%, with no significant difference between genders [2]. Upper airway obstruction during sleep in children with OSAHS results in decreased blood oxygen saturation and excessive blood carbonic acid content, which not only leads to cognitive impairment, excessive daytime sleepiness and inattention, but also tends to increase the risk of emotional instability and depression, thus affecting their health and leading to growth and development problems in children [3–5]. Polysomnography (PSG) is the gold standard for the diagnosis of OSAHS [6]. However, the detection rate of children’s PSG is not high, due to the limitations of PSG requiring admission monitoring, long time-consuming, cumbersome operation, high price and low child cooperation [7, 8]. Therefore, it is of great significance to research the biomarkers for screening OSAHS in children.

A novel type of small non-coding RNA, tRNA-derived fragments (tRF), has attracted our attention. tRNA-derived small RNAs fragments (tsRNA) are a class of small non-coding RNAs specifically cleaved from tRNAs with a length of 14–48 nucleotides [9]. Based on length and origin, they are divided into three main categories, namely tRNA fragments, stress-induced tRNA-derived RNA fragments (tiRNA), and toxic small tRNA-derived RNA fragments [10]. As the second most common small ncRNA after micro RNAs (miRNA), more and more data suggest that these ncRNAs have a wide range of biological functions, including cell and tissue stress response, tumorigenesis, immune response and so on [11]. Similar to miRNAs, stable stem loop structure, stable nucleoprotein complex and exosome encapsulation keep tRFs at a high concentration in serum [10]. tRFs in serum is sensitive to acute inflammation, aging, acute kidney disease and tissue injury, and may be a useful non-invasive biomarker [11].

Obesity is one of the important causes of OSAHS [12]. Research shows that the prevalence of OSAHS in obese children is 19% ~ 60% [13]. Like patients suffering from Obesity, the OSAHS patients would seem to be characterized by a chronic systemic inflammatory state [14, 15]. Chronic intermittent hypoxia leads to repeated hypoxia and reoxygenation cycles, enhances systemic oxidative stress, and leads to the production of systemic inflammation related biomarkers. Under specific conditions such as stress and hypoxia, tRNAs will be specifically cleaved into tsRNAs [9]. In recent years, it has been found that some small RNA molecules, including miRNA and circular RNAs (circRNA), are involved in the occurrence and development of OSAHS [16, 17]. According to all aforementioned findings, we assume that particular tRFs could be promising molecular biomarkers in OSAHS.

## Methods

### Patient sample collection and processing

This study recruited children with snoring as the main complaint in the Otolaryngology or Respiratory Department of Ningbo women’s and children’s Hospital from November 2020 to August 2021. Participants in the healthy control group were children of hospital colleagues. We performed a free PSG on all subjects. The diagnostic criteria of OSAHS were implemented in accordance with the Diagnosis and Treatment of Obstructive Sleep Apnea in Chinese Children guidelines (2020). Our exclusion criteria were children suffering from anemia, congenital heart disease, craniofacial deformities and other diseases. The study was approved by the ethics committee of Ningbo Women’s and Children’s Hospital (no.EC2020-047). Informed consents have been signed by the guardians of all subjects in this study.

Serum was collected according to the operating steps of the earlier research for serum and plasma collection. 1mL of peripheral venous blood samples were acquired before surgery and placed into an EDTA anticoagulant tube. All collected specimens should complete plasma separation within 1 h. After 10 min of centrifugation at 3000 rpm in 4 °C temperature, the 0.5ml plasma was transferred to new RNase-free tubes. The entire sample processing procedure was performed on ice. Finally, we froze the plasma at − 80 °C for further experiments.

### Total RNA extraction

In this study, according to the requirements of the commercial kit, a fixed volume of plasma (500 µl per sample) was used for tsRNA sequencing and quantitative reverse transcription-polymerase chain reaction (qRT-PCR) detection. Using TRIzol LS reagent (Invitrogen, Carlsbad, CA, USA), total RNA was extracted on the basis of the manufacturer’s instructions. Before proceeding with subsequent experiments, we measured the extracted RNA sample on NanoDrop® ND-1000 and confirmed that the absorbance ratio of all OD 260/280 was between 1.8 and 2.0. Finally, we stored all extracted RNA in a refrigerator at -80 °C for subsequent experiments.

### tsRNA pretreatment and high-throughput sequencing

In the initial study, we randomly selected plasma samples from 5 children with OSAHS and 5 healthy controls for high-throughput sequencing. There were amounts of redundant modifications on tsRNA that will interfere with the subsequent construction of small RNA libraries. Before small RNA library preparation, we firstly used rtStarTM tRF and tiRNA Pretreatment Kit (Arraystar, USA) to process total RNA. This step included the deacylation of 3’-aminoacyl (charged) to 3’-OH; 3’-cP (2’,3’-cyclic phosphate) was removed to 3’-OH for ligation with 3’adapter; 5’-OH (hydroxyl) phosphorylation to 5’-P for ligation with 5’adapter; and demethylation of m1A and m3C for achieving efficient reverse transcription. After this, following the instructions of the commercial kit NEB Next® Multiplex Small RNA Library Prep Set for Illumina (New England BioLabs, USA), and applied it to the pretreated total RNA. This procedure included the 3’ adapter ligation, the 5’ adapter ligation, cDNA synthesis and the preparation for library PCR amplification. Then, according to the requirements of kit, we denatured the prepared small RNA library into single-stranded DNA, captured it on the Illumina flow cell and amplified it in situ. Then we performed the cycle on the Illumina NextSeq 500 system.

### Transcriptome high-throughput sequencing data analysis

Sequence analysis was performed by the Solexa pipeline (Off-Line Base Caller software version 1.8, Illumina, Inc.). This step mainly included graphical analysis and basic calling. The detection of high-quality reads was performed by FastQC software version 0.11.5. Then, we used Novo Align software version 2.07.11 to compare the trimmed reads with the tRNA precursor sequence from GtRNAdb. Beyond this, other unrecognized reads were compared with several small RNA databases (such as mRNA, ribosomal RNA, small nuclear RNA, small nucleolar RNA, Piwi-interacting RNA and miRNA). The expression level of tsRNA was calculated according to the value of the number of normalized transcripts. The expression profile between the OSAHS patients and the healthy controls were compared by calculating the fold change of each tsRNA (the ratio of the group means). This work was done by Kangcheng Company (Shanghai, China). The analysis results revealed that a total of 11 tsRNAs showed significantly different expression (Table 1). On this basis, we further selected a tsRNA with fold change ≥ 2, *P* value < 0.05 and significant differences between the case group and the control group for subsequent experiments, namely tRF-21-U0EZY9X1B (tRF-21).

**Table 1:** tsRNAs with significant difference between the two groups

tRF_ID	MINTbase_ID	Fold_Change	*p*_value
tRF-51:71-chrM.Pro-TGG	tRF-21-U0EZY9X1B	213.5216156	0.048734815
tRF-1:29-Gly-TCC-1	tRF-29-QNR8VP9NFQEW	125.2706859	0.036281415
tRF-1:22-chrM.Ser-GCT	tRF-22-5BF900BY3	0.001450162	0.001531281
tRF-1:28-chrM.Ser-TGA	tRF-28-OB1690PQR304	0.001800344	0.003122924
tRF-1:14-Gly-CCC-2	/	0.002482395	0.00926424
tRF-1:22-Gly-CCC-2	tRF-22-Q1Q89P9L5	0.003703841	0.028273208
tRF-1:29-chrM.Ser-TGA	/	0.003761660	0.029452493
tRF-1:16-Val-CAC-3	tRF-16-79MP9PD	0.003868279	0.031385244
tRF-67:85-Ser-AGA-1-M6	tRF-19-DRXSE5I2	0.003882877	0.031846161
tRF-1:15-Glu-CTC-1-M4	/	0.004223015	0.038933891
tRF-1:14-Gly-CCC-1-M5	/	0.012718105	0.044981046

### Quantitative RT-PCR

**Fig. 1:**
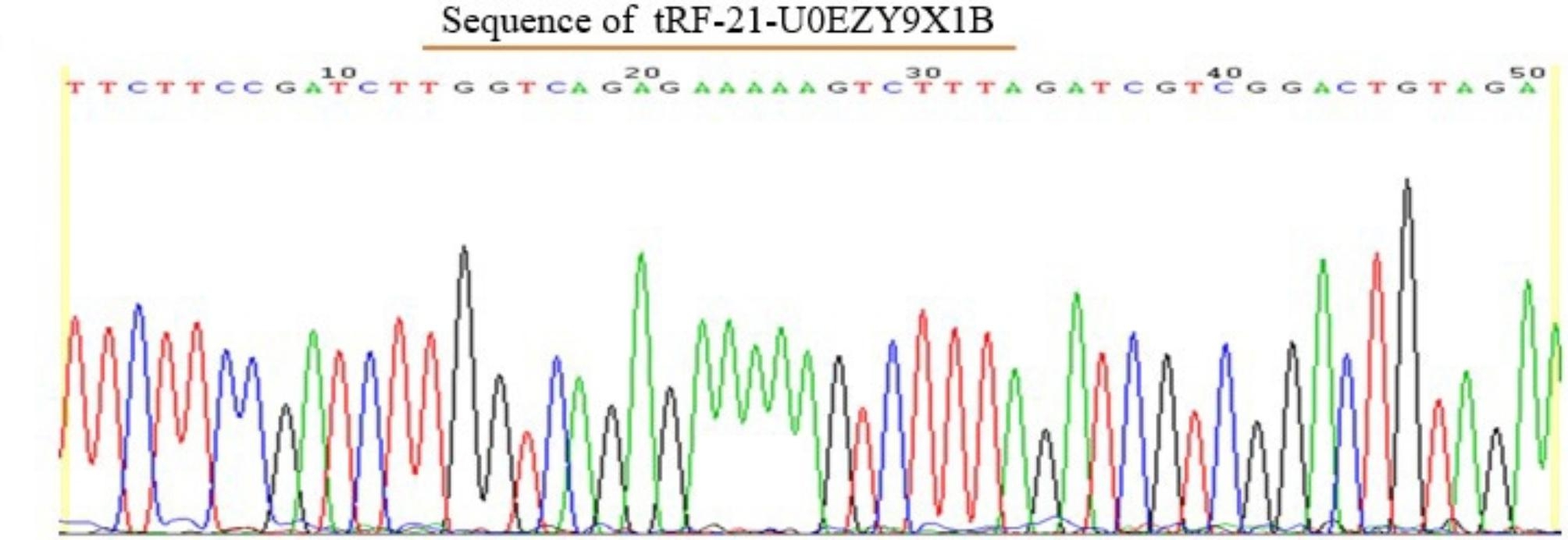
Sequence results of qRT-PCR products of tRF-21-U0EZY9X1B

qRT-PCR is considered to be the gold standard for gene expression quantification in various studies. Firstly, we performed qRT-PCR on the plasma samples of 5 children with OSAHS and 5 healthy participants who completed high-throughput sequencing. The comparison results of the sequencing of products showed that the amplification primers were specific (Fig. 1). Then, we detected the expression level of tRF-21 in plasma samples of other subjects to verify the sequencing results of tsRNA. The rtStarTM tRF and tiRNA Pretreatment Kit (Arraystar, USA) was used to pretreat modifications of RNA samples, and rtStar™ First-Strand cDNA Synthesis kit (Arraystar, USA) was used to reverse transcribe pretreated RNA into cDNA. qRT-PCR was performed using GoTaqqPCR Master Mix Kit (Promega, USA) with specific primers (F: 5’CTACAGTCCGACGATCTAAAGA3’; R: 5’TCTTCCGATCTTGGTCAGAG3’). The study used U6 (F: 5’GCTTCGGCAGCACATATACTAAAAT3’; R: 5’CGCTTCACGAATTTGCGTGTCAT 3’) as an internal reference for evaluating efficiency. Three replicate experiments were performed on each specimen.

### Statistical analysis

The ΔCt method was used for data analysis of qRT-PCR. It should be noted that the higher the ΔCT value, the lower the actual expression of tRF in serum. Chi-square test was used to compare the age between the two groups, and Student’s t test was used for other basic information and clinical indicators. The expression of tRF-21 between the two groups was compared by two independent sample t-test, and the regression analysis was performed by linear regression (Backward estimation). Data with a *p* value < 0.05 was considered statistically significant. All the calculations mentioned above were conducted by SPSS v.20.0 and GraphPad Prism v.8.0.

## Results

### Participants’ characteristics

In this study, 50 and 38 children were enrolled in the case group and the control group respectively. Demographic characteristics of all subjects are summarized in Table 2. In addition to OAHI and LaSO2, there were significant differences in height, serum creatinine (Scr) and total cholesterol (TC) between the two groups.

**Table 2:** Demographic information and laboratory parameters of subjects

Parameter	case group (n = 50)	control group (n = 38)	p value	t/χ^2^	95% CI
Gender (male / female)	27 / 23	22 / 16	0.716	0.133	-
Age (years)	5.35 ± 0.81	5.63 ± 0.75	0.099	-1.667	-0.617–0.054
Height (cm)	111.89 ± 6.60	115.41 ± 9.38	0.042	-2.065	-6.905 - -1.307
Weight (kg)	20.78 ± 4.60	21.34 ± 4.15	0.556	-0.592	-2.451–1.327
BMI (kg/m^2^)	16.50 ± 2.47	15.93 ± 1.80	0.233	1.201	-0.374–1.514
OAHI (events/h)	8.94 ± 4.56	0.18 ± 0.13	< 0.0001	11.837	7.290–10.233
LaSO2 (%)	70.68 ± 14.63	90.58 ± 3.42	< 0.0001	-8.206	-24.719 - -15.078
Hb (g/dL)	12.39 ± 0.95	12.72 ± 0.77	0.101	-1.661	-0.732–0.066
MCH (pg)	27.71 ± 1.77	27.80 ± 1.26	0.807	-0.245	-0.809–0.632
Plt (10^9^/L)	334.45 ± 84.55	326.53 ± 62.04	0.650	0.455	-26.695–42.530
AST (U/L)	30.60 ± 6.66	28.66 ± 5.58	0.150	1.451	-0.718–4.602
ALT (U/L)	12.91 ± 6.20	11.87 ± 3.99	0.368	0.904	-1.251–3.338
UA (µmol/L)	260.05 ± 55.99	277.42 ± 62.25	0.173	-1.373	-42.514–7.772
Scr (µmol/L)	50.97 ± 9.69	45.82 ± 9.96	0.017	2.442	0.958–9.347
BUN (mmol/L)	4.96 ± 1.34	5.12 ± 1.34	0.569	-0.572	0.738 − 0.408
Glucose (mmol/L)	5.14 ± 0.73	5.13 ± 0.91	0.922	0.099	-0.330–0.365
TG (mmol/L)	1.21 ± 0.59	1.01 ± 0.43	0.095	1.690	-0.034–0.417
TC (mmol/L)	4.60 ± 0.66	4.29 ± 0.59	0.024	2.306	0.043–0.581
CK-MB (U/L)	25.43 ± 4.48	24.36 ± 8.59	0.451	0.757	-1.742–3.882

CI: confidence interval; BMI: body mass index; OAHI: obstructive apnea hypopnea index; LaSO_2_: lowest oxygen saturation; Hb: hemoglobin; MCH: mean corpuscular hemoglobin; Plt: platelet; AST: aspartate aminotransferase; ALT: alanine aminotransferase; UA: uric acid; Scr: serum creatinine; BUN: blood urea nitrogen; TG: triglyceride; TC: total cholesterol; CK-MB: creatine kinase-MB

### Expression profiles of tsRNAs differ among OSAHS children and healthy children

**Fig. 2:**
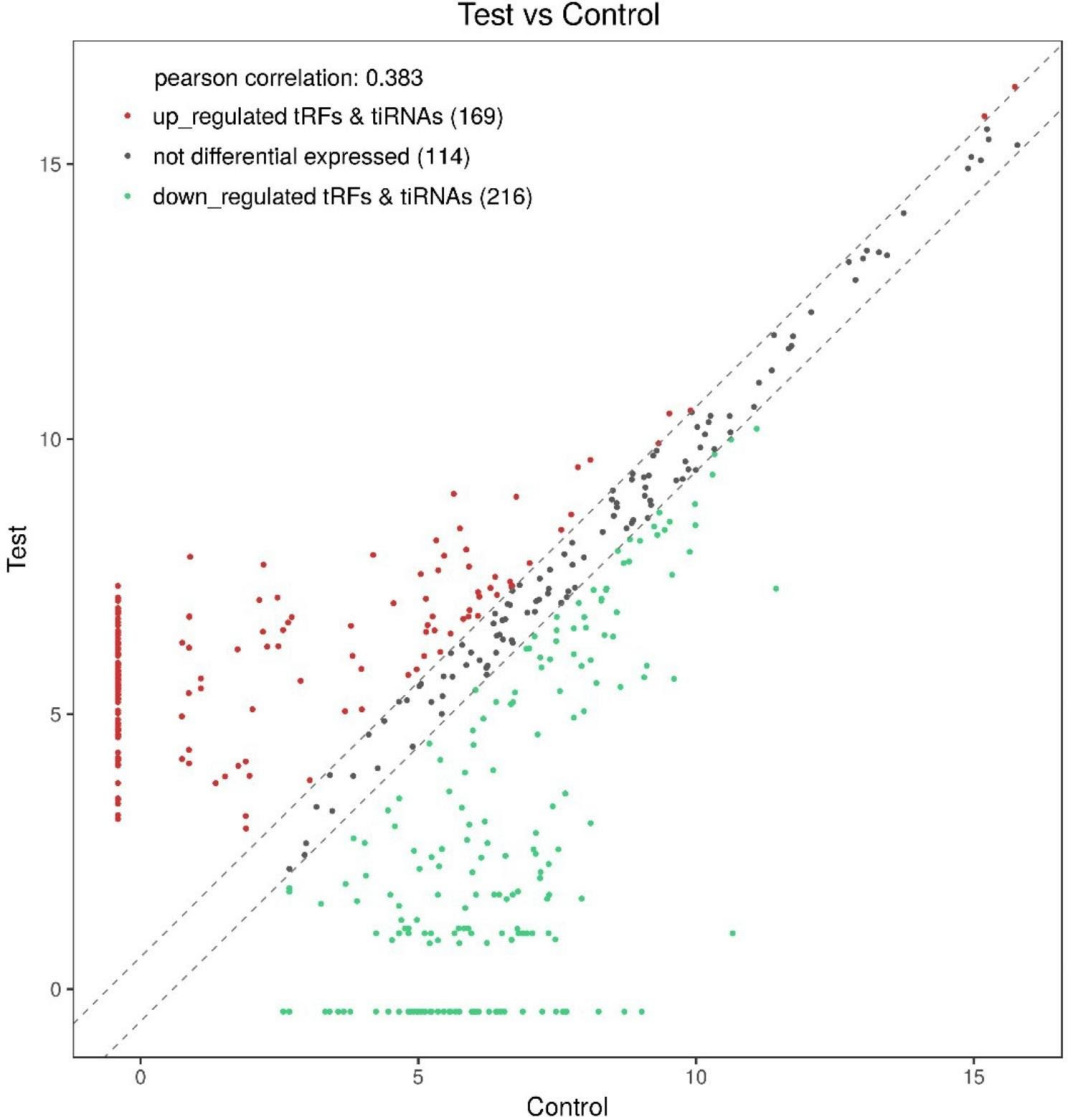
Differentially expressed genes for tsRNA levels in plasma of 5 healthy people and 5 children with OSAHS. The values of X and Y axes in the scatter plot are the averaged CPM values of each roup (log_2_ saled). tsRNAs above the top line (red dots, up-regulation) or below the bottom ine (green dots, down-regulation) indicate more than 1.5 folds change between the two compared groups. ray dots indicate non-differentially expressed tsNAs.

**Fig. 3:**
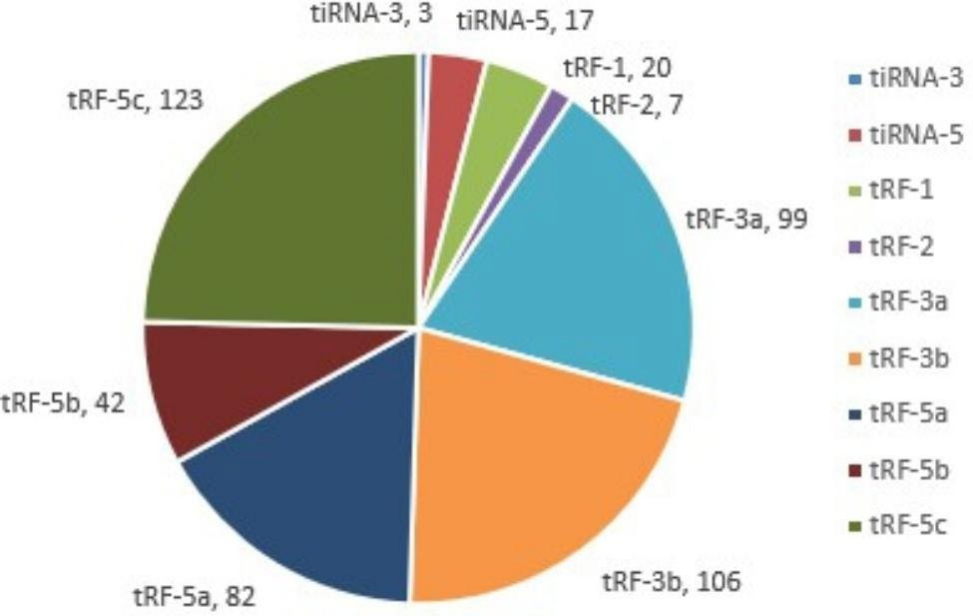
The Pie showed the tsRNA subtypes. Different colors represented the numbers of tsRNA subtypes and the number of subtypes was marked next to it

In order to explore the function of small molecule tsRNA in OSAHS disease, we selected plasma samples of 5 OSAHS children and 5 healthy individuals for high-throughput sequencing. Transcriptome high-throughput sequencing results detected that there were 499 differentially expressed tsRNAs between OSAHS children and healthy controls (Fig. 2), of which 169 tsRNAs showed up-regulation and 216 tsRNAs showed down-regulation. Among these differentially expressed tsRNAs, the most abundant subtype was tRF-5c, with the number of 123, and the proportion of tiRNA-3 subtypes was the smallest (Fig. 3). Notably, between the two groups, there was a significant difference of at least 5 times in the expression of 11 tsRNAs (fold changes ≥ 5.0; *P* < 0.05) (Table 2). Two tsRNAs were up-regulated and the other 9 tsRNAs are down-regulated. In this study, we chose tRF-21 as our target research object. We successfully designed specific primers for tRF-21, and the sequencing results of the qRT-PCR products showed that they completely consistent with the original sequences (Fig. 1).

### Differential expression and predictive value of tRF-21

**Fig. 4:**
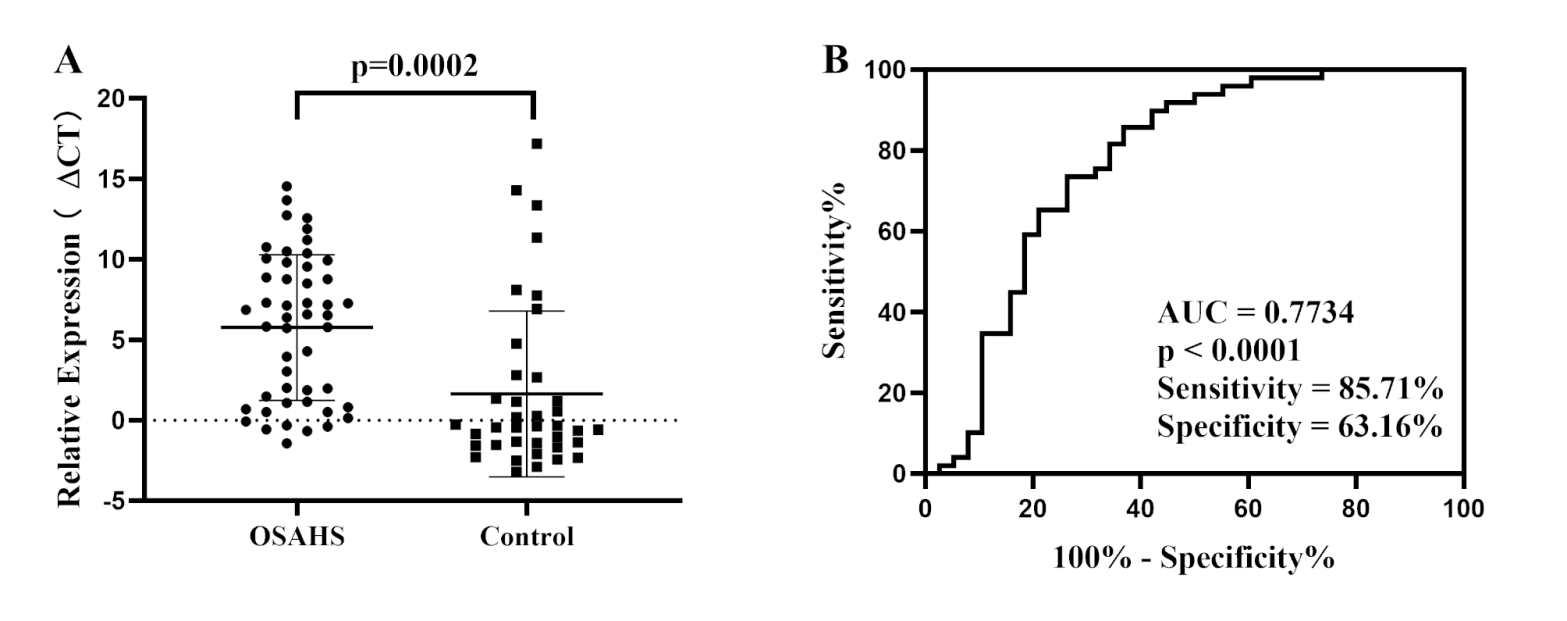
Differential expression and diagnostic value of tRF-21-U0EZY9X1B. Student t-test of detecting the expression difference of tRF-21-U0EZY9X1B between the two groups (**A**) (t = 3.969, 95% CI: -6.194 to -2.059); Receiver operating characteristic curve of tRF-21-U0EZY9X1B (**B**) (95% CI: 0.666 to 0.881)

We detected the expression of tRF-21 in all collected serum samples. The results of student t-test show that the ΔCT of OSAHS group is obviously higher than that of healthy control group (p = 0.0002), which means that the serum expression level of tRF-21 in OSAHS children decrease significantly (Fig. 4A). As shown in Fig. 4B, the area under the receiver operating characteristic (ROC) curve of tRF-21 is 0.7734. The sensitivity and specificity are 85.71% and 63.16%, respectively. These results suggest the potential value of tRF-21 in the screening of OSAHS.

### The relationship between levels of tRF-21 in plasma and clinical parameters

**Fig. 5:**
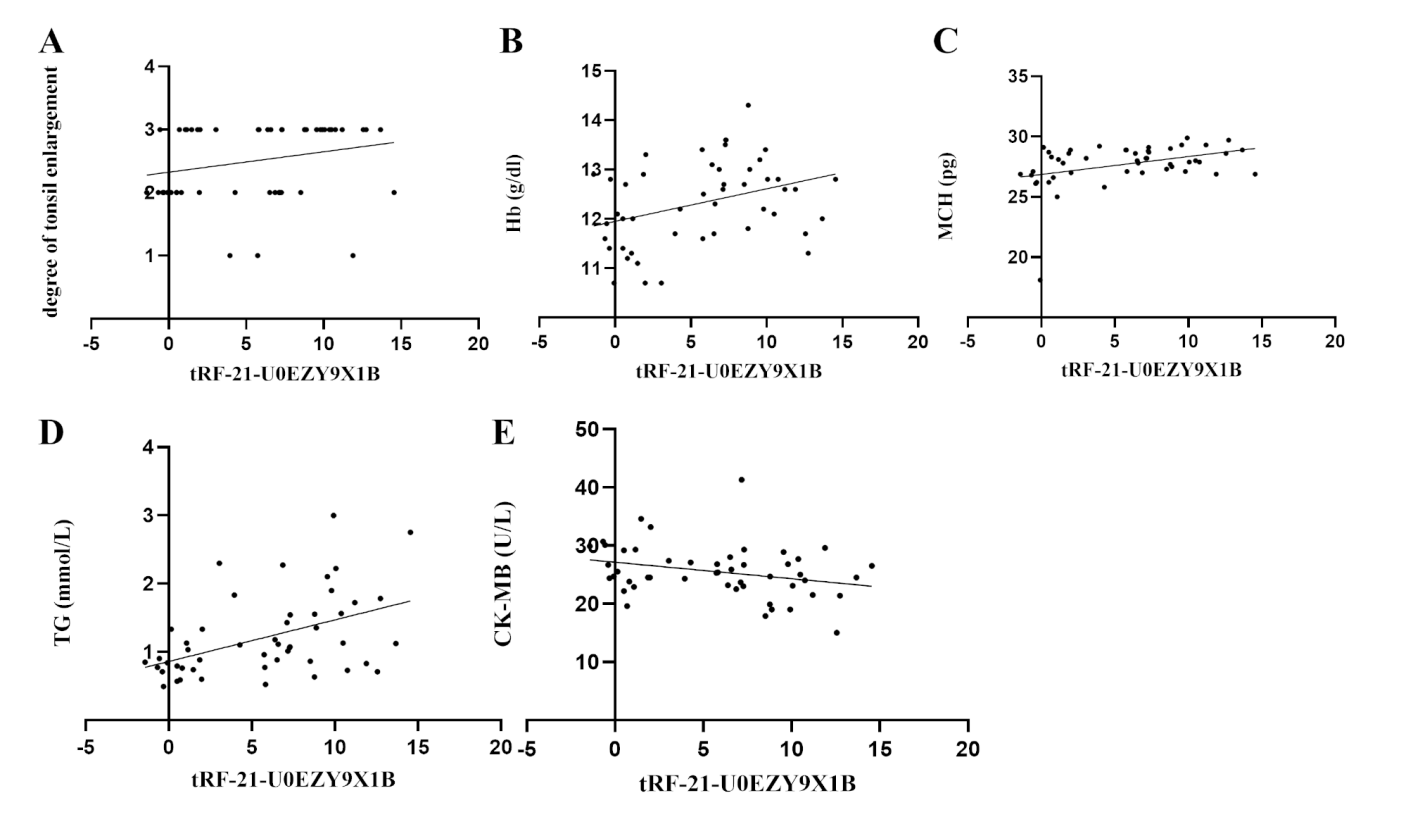
Scatter plot of correlation between tRF-21-U0EZY9X1B and degree of tonsil enlargement (**A**), Hb (**B**), MCH (**C**), TG (**D**) and CK-MB (**E**)

In addition to the parameters in Table 1, we also collect the degree of tonsil enlargement, which is divided into grade I, II and III, with 3, 19 and 28 children respectively. Spearman correlation is used to analyze the correlation between the degree of tonsil enlargement and tRF-21, and Person correlation is used for other parameters. The results show that the level of plasma tRF-21 is significantly correlated with the degree of tonsil enlargement, Hb, MCH, TG and CK-MB (Table 3). Figure 5 shows that the tRF is basically linear with the above five factors.

**Table 3:** The relationship of tRF-21-U0EZY9X1B levels (∆Ct) in plasma with clinical parameters of childrens with OSAHS

Parameters	Correlation coefficient	P value
Age	-0.008	0.954
Height	-0.014	0.922
Weight	0.073	0.620
BMI	0.100	0.492
OAHI	-0.146	0.318
LaSO2	0.042	0.772
Degree of tonsil enlargement	0.337	0.018
Hb	0.351	0.015
MCH	0.374	0.009
Plt	-0.028	0.850
AST	0.071	0.628
ALT	0.189	0.192
UA	0.084	0.567
Scr	0.134	0.358
BUN	-0.169	0.247
Glucose	0.029	0.844
TG	0.465	0.001
TC	-0.054	0.711
CK-MB	-0.286	0.047

Interestingly, the expression level of tRF-21 can be predicted by MCH, TG and CK-MB together (ΔCt = 0.684*MCH + 2.796*TG – 0.245*CK-MB), which is revealed by multivariable linear regression model (Table 4).

**Table 4:** Multivariate linear regression analysis of the expression level of tRF-21-U0EZY9X1B

Associated parameters	B	SE	95% CI	t	p value
Degree of tonsil enlargement	0.083	0.923	-1.259–2.467	0.655	0.519
Hb	0.176	0.711	-0.499–2.372	1.317	0.198
MCH	0.684	0.317	0.045–1.324	2.158	0.036
TG	2.796	0.956	0.868–4.723	2.923	0.005
CK-MB	-0.245	0.123	-0.493–0.002	-1.996	0.052
Constant	-10.412	9.307	-29.169–8.345	-1.119	0.269

SE: Standard Error; CI: Confidence Interval

## Discussion

OSAHS can occur at all ages, but the peak onset period in children is 4 to 7 years old because of the tonsils and adenoids accounting for the largest proportion of airway volume at this time. Therefore, we recruited children between the ages of 4 to 7 for this study. Interestingly, we observed that children with OSAHS had significantly lower height and higher TC and Scr than the control group (Table 1). A case-control study found that underweight children with OSAHS, as compared with those without OSAHS, were more likely to have decreased height and the height was negatively correlated with OSAHS [18]. Obesity is a recognized risk factor for OSAHS. However, in our study, there was no difference in weight and BMI between the two groups, but the TC of children in the OSAHS group was significantly higher than that in the control group. Several studies have shown that the TC of adolescents and middle-aged OSAHS patients was significantly increased, which is the same as our results [19–21]. OSAHS contributes the impairs of renal function [22, 23]. A study on the relationship between OSAHS and renal function found that there were varying degrees of impairment of renal function in OSAHS patients, but there was no statistically significant difference in Scr between the OSAHS group and the control group [24].

In this study, we first performed high-throughput sequencing to detect the expression profile of plasma tsRNAs in 5 OSAHS children and 5 control children. According to the sequencing data, tRF-21 with the most significant difference was selected for qRT-PCR to verify the authenticity of the profile. The comparison results of the sequencing of products showed that the amplification primers were specific (Fig. 1). Then, we detected the expression level of tRF-21 in the plasma of all 50 children in the case group and 38 children in the control group. The results showed that tRF-21 was significantly down regulated in children with OSAHS compared with healthy controls (Fig. 4). tRF-21 is usually studied and explored as a tumor biomarker, such as pancreatic ductal Adenocarcinoma [25], papillary thyroid cancer [26], cholangiocarcinoma [27] and lung adenocarcinoma [28]. Pan et al. found that tRF-21-VBY9PYKHD, as an inflammatory cytokine–regulated transfer fragment has a tumor-suppressive effect [25]. In addition to tumor inhibition, other effects of tRF-21 have not been understood.

Previous studies have reported that OSAHS is a disease with chronic systemic inflammation and metabolic changes [29, 30]. OSAHS, through intermittent hypoxia and sleep fragmentation, was involved with sympathetic activation, endothelial dysfunction, inflammatory state, and oxidative stress with multi-organ involvement and comorbidities, in particular, such as cardiovascular, cerebrovascular and neoplastic [31]. A research by Santamaria-Martos et al. found that patients with OSAHS exhibit a dysregulated miRNA profile compared to healthy controls [32]. It appears that intermittent hypoxia associated with OSAHS, could lead to differential expression of some hypoxia-induced miRNAs. Another study reported that double-stranded RNA composed of a tRF and its complementary sequence significantly promoted the survival of myocardial cells after hypoxia/reoxygenation in vitro [33]. There are few studies on the relationship between tRF and hypoxia or sleep disorders, which is also one of the future research directions. The relationship between tRFs and hypoxia or sleep disorders needs more researches in the future.

ROC curve showed that the area under the curve of tRF-21 was 0.7734, and the sensitivity and specificity were 85.71% and 63.16% respectively. These results suggest the potential value of tRF-21 in OSAHS screening in children. At present, studies on OSAHS in children have not obtained clear biomarkers, and existing studies also have some limitations, such as lack of specificity and sensitivity analysis [34]. As a screening method, the tRF has the characteristics of easy sample acquisition and convenient laboratory operation, which can be regarded as a new research direction. Of course, it requires further validation of the results in a larger cohort.

The results of correlation analysis showed that the expression level of tRF-21 was linearly correlated with the degree of tonsillar enlargement. It suggests that the level of tRF-21 may be related to the severity of OSAHS. However, there is no significant correlation between the tRF and the OAHI index. In addition, the tRF-21 level is significantly correlated with Hb, MCH, TG and CK-MB, which could be predicted by MCH, TG and CK-MB. At present, the research on the function of tRF-21 is very limited.

In conclusion, our study found that the plasma tRF-21-U0EZY9X1B level of 4 to 6-year-old OSAHS children was down regulated, which was closely related to Hb, MCH, TG and CK-MB, and could be used as a potential biomarker for the diagnosis of OSAHS in children. However, our research has some limitations, including the problem of insufficient sample size. In the future, we will verify this result in a larger cohort.

## Electronic supplementary material

Below is the link to the electronic supplementary material.

Supplementary Material 1

## Data Availability

All raw data have been uploaded to NCBI database (BioProject ID: PRJNA862251). https://www.ncbi.nlm.nih.gov/bioproject/PRJNA862251/.

## Abbreviations

OSAHS: Obstructive sleep apnea hypopnea syndrome
PSG: Polysomnography
tRF: tRNA-derived fragment
tsRNA: tRNA-derived small RNAs fragment
tiRNA: tRNA-derived RNA fragment
miRNA: Micro RNA
circRNA: Circular RNA
qRT-PCR: Quantitative reverse transcription-polymerase chain reaction
ROC: Receiver operating characteristic
OAHI: Obstructive apnea hypopnea index
LaSO_2_: Lowest oxygen saturation
Scr: Serum creatinine
BUN: Blood urea nitrogen
TG: Triglyceride
TC: Total cholesterol
CK-MB: Creatine kinase-MB
