# Supplementary material for: The potential of tRF-21-U0EZY9X1B plasmatic level as a biomarker of children with obstructive sleep apnea-hypopnea syndrome
Source: BMC Pediatr. 2023 Apr 26;23:197. doi: 10.1186/s12887-023-04020-2 (PMC10134554; doi:10.1186/s12887-023-04020-2)
Supplement: Supplementary file 1 — Supplementary Material 1 [file 12887_2023_4020_MOESM1_ESM.docx]

Supplementary table 1 Demographic characteristics of 10 children in the pre-experiment

| Parameter | case group | control group | p value | t/ꭓ^2^ | 95% CI |
| --- | --- | --- | --- | --- | --- |
|  | (n=5) | (n=5) |  |  |  |
| Gender (male / female) | 3 / 2 | 3 / 2 | 1.000 | 0.000 | - |
| Age (years) | 5.53±1.02 | 5.54±0.85 | 0.982 | -0.024 | -1.37 - 1.35 |
| Height (cm) | 111.60±4.39 | 111.30±7.73 | 0.942 | 0.075 | -8.87 - 9.47 |
| Weight (kg) | 21.70±2.54 | 19.56±1.32 | 0.133 | 1.672 | -0.81 - 5.09 |
| BMI (kg/m^2^) | 17.38±1.26 | 15.89±1.73 | 0.158 | 1.557 | -0.72 - 3.69 |
| OAHI (events/h) | 11.04±5.40 | 0.14±0.05 | 0.002 | 4.516 | 5.34 - 16.47 |
| LaSO2 (%) | 65.40±16.09 | 92.40±1.52 | 0.006 | -3.736 | -43.66 - -10.34 |
